# Persisting high rates of mental health disorders in patients in opioid agonist treatment—Results from a 6‐year longitudinal study

**DOI:** 10.1111/ajad.70085

**Published:** 2025-09-14

**Authors:** Michael Soyka, Gabi Koller, Wittchen Hans‐Ulrich, Gerhard Bühringer

**Affiliations:** ^1^ Department of Psychiatry and Psychotherapy, Psychiatric Hospital University of Munich Munich Germany; ^2^ Department of Psychology Technical University of Dresden Munich Germany; ^3^ Institut für Therapieforschung LMU University Hospital, LMU Munich München Germany

## Abstract

**Background and Objectives:**

There are hardly any data from longitudinal studies on the prevalence of mental health disorders in patients in opioid agonist therapy (OAT).

**Methods:**

Here we report prevalence rates of mental health disorders in a 6‐year naturalistic noninterventional follow‐up study of patients in OAT (*N* = 2694 at baseline).

**Results:**

The number of participants without any psychiatric diagnosis only modestly increased from 36.7% to 42.2% (males 42.6%, females 47.6%) over a 6‐year period. Depression (42%), anxiety disorders (19.3%), sleep disorders (21.3%), and posttraumatic stress disorder (13%) were most frequent. The rates for patients in psychiatric or psychological/psychotherapeutic treatment at baseline were rather low (8% resp. 8–12%) and declined over time (3.6% resp. 5.4%–6.8% after 6 years).

**Conclusions and Scientific Significance:**

Data from this long‐term study indicate an overall high persisting prevalence of mental health disorders in patients in OAT and a rather low number of patients in psychiatric/psychological treatment. Hence, this study indicates a substantial need for specific psychosocial and psychopharmacological interventions in patients in opioid agonist therapy.

## INTRODUCTION

Opioid use disorder (OUD) is seen as a chronic relapsing disorder. In 2017, worldwide 40.5 million people were dependent on opioids and 109,500 people died from opioid overdose.^1^ The global age‐standardised prevalence rate for opioid use disorder is about 0.5%. Frequent comorbid mental health disorders are brain damage (encephalopathy, injuries, others), depression and anxiety, as well as suicide.^2^

Worldwide opioid agonist treatment (OAT) is the first‐line treatment in OUD. First line medications are methadone and buprenorphine^3^ with proven efficacy to suppress opioid withdrawal symptoms, craving and relapse in opioid users.^2^, ^3^ OAT also reduces the risk of criminal behavior and improves psychosocial functioning and health outcomes in OUD.^3^ Second line pharmacological options are retarded morphine or diacetylmorphine.

Previous research has indicated a high comorbidity of substance use disorders with mental health disorders.^4^ High rates have especially been found in women and opioid users with a young age at first opioid use.^5^ Data from randomized clinical studies indicate a high comorbidity with mental health disorders to be associated with worse substance use outcomes and poorer psychosocial functioning.^6^ There are few longitudinal studies on the prevalence rates of mental health disorders in OAT patients. Comorbid mental health disorders may have a significant impact on compliance, mortality, well‐being, and psychosocial integration of patients in OAT. In addition, comorbid disorders in patients in OAT have a significant and negative impact on quality of life.^7^, ^8^, ^9^ Unfortunately, there is a lack of long‐term longitudinal studies on this subject to clarify the potential need for psychosocial interventions.

Here, we report a secondary analysis of data concerning psychiatric comorbidity in a sample of 1694 patients in OAT.

## METHODS

In brief, methods and assessments used in this study have been reported in detail elsewhere.^10^, ^11^ This is a secondary analysis of data from an observational 6‐year naturalistic prospective longitudinal study in an unselected prevalence sample of patients in opioid agonist treatment. The study was strictly observational and the physicians providing OAT were free to decide on individual treatment, medication and further treatment.

The patients included were a nationally representative sample.^11^ The study (data collection) was conducted from 2004 to 2010. Assessments included a comprehensive baseline examination (T‐0), and clinical assessments at 12‐month (T‐1) and at 72‐month (T‐3) follow‐up which consisted of a self‐report patient questionnaire, urine drug tests and a comprehensive clinical interview and treatment documentation by the patients physician (for details see 11).

Inclusion criteria: Patients aged at least 16 years with current opioid dependence (ICD‐10 criteria for opioid dependence) in opioid agonist therapy with either buprenorphine or methadone were eligible for the study. Those two medications were approved for treatment opioid use disorder in Germany at the start of the study.

Exclusion criteria included acute medical disorders requiring treatment, severe cognitive impairment, and unwillingness to comply with study procedures. Written informed consent was obtained.

### Study participants

223 physicians agreed to participate in this study. At baseline a total of *N* = 2694 patients were enrolled.

### Assessment

The patient questionnaire consisted of various components such as item groups of the EuropASI and modules of the substance use questions of the WHO Composite International Diagnostic Interview (CIDI; for references see 11). This is a comprehensive and fully standardized diagnostic interview for assessing mental disorders containing 276 symptom questions.

The patient questionnaire covered different domains, including basic social and socio‐demographic information, past and current drug use and illness history module (CIDI), mental health and substance use diagnostic status (DSM‐IV substance use and other mental disorders by CIDI), among many others not reported here.

### Clinical interview and assessment

Each patient was evaluated by his doctor with a standardized interview that covered (i) current and past opioid maintenance treatments along with documentation of onset or discontinuation of all lifetime treatment episodes (e.g., dosage, dosing status), (ii) licit and illicit substance use behaviors and substance use (iii) past and current physical and mental resp psychiatric disorders, among others.

### Measures

Data about retention, drug use, or abstinence and concomitant psychiatric or somatic diagnoses were based on the treating doctor's rating and assessment in the follow‐up interviews.

### Statistical procedures

Analyses were conducted in Stata SE, version 12.1.^8^, ^9^

## RESULTS

Of the 2694 baseline patients 1624 (1100 male, 524 female, mean age 41 years at follow‐up) were assessed for outcome at the final comprehensive 72‐month follow‐up assessment (T‐3). Mean duration of opioid use was 15.4 years. 21.4% of patients at follow‐up used opioids, 38% used other drugs including benzodiazepines (22.5%) or cocaine (8.3%). 59.5% of patients were tested positive for some form of substance use including cannabis.131 patients had died during the 6‐year follow‐up period.^11^

The analyses are based on patients who were still in treatment at the 72‐month follow‐up assessment (i.e., excluding those who were not retained in treatment, had died or became abstinent) and for whom complete baseline and follow‐up documentation was available.

For outcome at T3, *n* = 1216 were treated with methadone, *n* = 270 with buprenorphine, and *n* = 7 with other drugs. In 1139 patients no change of medication had been performed over the whole 6 year period: Full information was available for 943 patients treated with methadone, 196 with buprenorphine. 208 patients completed the opioid maintenance treatment within the study period, either because they became drug‐free or were referred to an abstinence‐oriented treatment setting.

### Baseline characteristics

**Figure 1:**
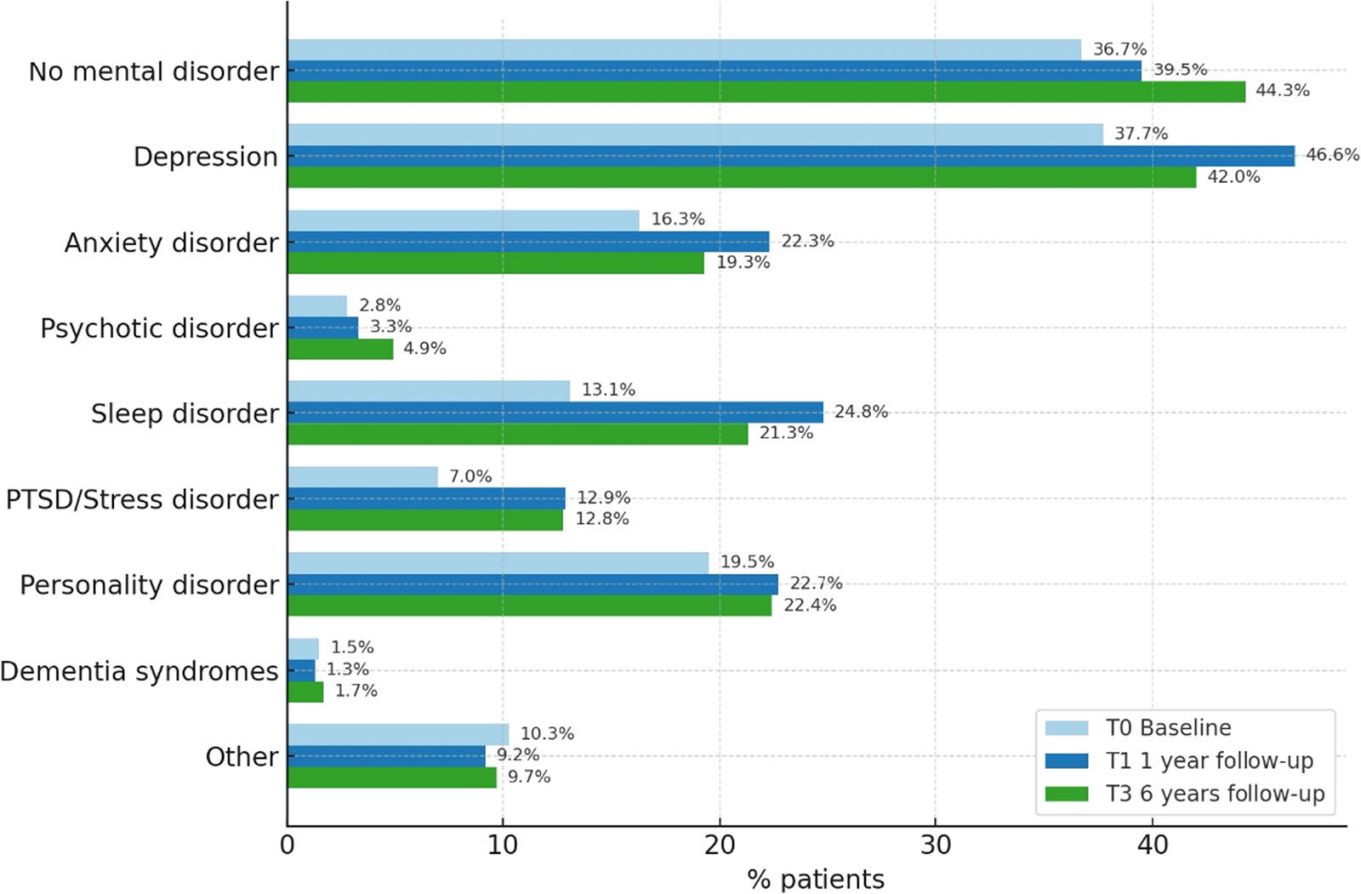
Prevalence of mental health disorders over time.

Baseline characteristics and initial findings on mental health disorders of this sample at baseline have been previously reported.^11^ In brief, 2694 individuals were included. 68.4% received at least one psychiatric diagnosis other than substance use. 53% had one, 28.5% two, 12.5% had three and 5.9% 4 or more diagnosis. (see Figure 1). Depression (56.6%) and anxiety disorders (25.3%) were the most frequent diagnoses.

EUROP ASI composite scores at baseline were 2.8 (SD 1.5) for the methadone group, 2.3 (SD 1.2) for the buprenorphine group (*p* < .000). Mean ASI scores at follow‐up were 2.2 in the methadone group versus 2.0 in the buprenorphine group.^9^ Mean dosages at baseline were 73.5 mg for methadone and 7.1 mg for buprenorphine.

### Psychiatric diagnoses

The number of patients without psychiatric comorbidity increased over the 6 year period modestly from 36.7% to 44.3% (*p* < .000). Most frequent diagnoses were depression (increase from 37.7% to 42% at T‐3, *p* = .001), anxiety disorders (16.3% to 19.3%, *p* < .000) and sleep disorders (13.1% to 21.3%, *p* < .000). Psychotic disorders (2.8% to 4.9%, *p* < .000) were much rarer. Figures for posttraumatic stress disorder increased from 7.0% to 13.0% (*p* < .000) 34.4% of the participants at T‐1 but 26.4% at T‐3 had at least one psychiatric diagnosis, 17.0% (13.5%) two, 8.3% (8.0%) three, and 3.7% (7.8%) four or more diagnoses for mental disorders. 37.5% of males at T‐1 but 42.6% at T‐3 did not meet the criteria for any psychiatric diagnosis, compared to 35.1% of females at T‐1 (47.6% at T‐3).

Psychosocial and other treatment: At baseline (T‐0) 8% of patients were in psychiatric treatment, only 3.6% at follow up after 3 years (T‐3). Figures for other forms of treatment also declined over time: psychological treatment from 12.% to 5.4%, psychotherapy from 8% to 6.8%, social services from 14.3% to 8.2%. The average number of visits in psychiatric or psychological practice/services in the last 12 months prior T‐0 or T‐3 were consistenly low over time: 0.8 versus 0.9 for psychiatrists, 0.9 versus 1.3 psychologists—but 7.3 resp. 9.9 for other nonpsychiatric medical treatments.

## DISCUSSION

This longitudinal noninterventional 6‐year follow‐up study in 2694 individuals in opioid agonist treatment indicates a persisting high prevalence rate of mental health disorders with an only modest decline over a 6‐year period. According to clinical diagnoses at baseline almost two‐thirds of the sample had at least one nonsubstance use disorder. The number of patients with comorbid psychiatric diagnoses decreased over time but many patients still met the criteria for more than one disorder. With respect to specific diagnosis, the most frequent mental disorders were depression (37%–43%), anxiety disorders (16%–19%), and sleep disoders (13%–21%) which all slightly increased over time. Most of the affected patients met the criteria for two or more disorders, with few differences between males and females.

The data overall of this naturalistic noninterventional study indicate a persistent high comorbidity with mental health disorders in patients in opioid maintenance treatment over a 6‐year period. The overall retention to treatment in this study was excellent.^11^ A high comorbidity with mental disorders was also found in other studies. Naji et al.^5^ reported that of 667 patients in methadone treatment 57.7% were diagnosed with a psychiatric disorder, predominantly anxiety disorders (33.8%). A high rate of lifetime mental disorders was also found in a randomised study comparing the effects of methadone and buprenorphine‐naloxone in a 24‐week trial in 1269 patients (595 completers). The rates for bipolar disorder were 28.3%, 22.3% for major depressive disorder, 50.8% for any anxiety disorder.^9^ Mood disorders (60%) and anxiety disorders (46%) were also frequent in methadone maintained patients studied by Carpentier et al.^7^ Patients with comorbid opioid use and mental disorders in the buprenorphine‐naloxone group reported greater reductions in the opioid use than treatment with methadone. A rather low number of individuals with psychotic disorders in OAT has also been found in other comprehensive studies.^12^ The rates of patients in psychiatric or psychological/psychotherapeutic treatment in this study were low and declined over time despite persisting high rates of mental health disorders. Data of this longitudinal study basically confirm data from cross‐sectional or short‐term studies.

Limitations: Since this was a noninterventional and not a controlled randomized study a number of critical issues can be raised. First, no control group of opioid dependent patients not in OAT was included. Second, no defined psychiatric interventions were mandatory or offered during treatment and the severity of the psychiatric disorders reported could not be adequately measured. Diagnoses were solely based on self‐report questionnaires and clinical diagnosis made by the treating physician but taking the long observation period into account the reported prevalence rates are plausible. Since this was a noninterventional study no treatment recommendations or interventions were provided. Treatment was solely based on the clinical decisions made by the treating physician and availability of treatment. Patients receiving treatment for mental health disoders were not excluded from this analysis.

In conclusion, data from this study indicate that mental health disorders, especially affective disorder and anxiety disorder, are very frequent in patients in opioid agonist treatment and figures for psychiatric comorbidity only modestly drop over a 6‐year period. Thus, OAT alone does not reduce rates of mental health disorders. The study underscores the necessity to provide adequate psychiatric care for these patients. The efficacy of integrated care models that adress both opioid use disorders and psychiatric comorbidities must be studied in more detail.

Future studies may focus on the efficacy of defined psychiatric and psychopharmacological interventions in OAT patients with comorbid mental health disorders. As data show, there is broad room for improvement in this area.

## CONFLICT OF INTEREST STATEMENT

For the past 5 years, M. S. has worked as a consultant for Camurus and Ethypharm.

## ETHICS STATEMENT

The study protocol was approved by the ethics committee of the Medical Faculty, Technical University of Dresden, Germany (EK 15022004, 21.4.2004).
